# Development and Validation of a Predictive Model for Occult Liver Metastasis in Pancreatic Ductal Adenocarcinoma Using Subjective Imaging and Clinical Data

**DOI:** 10.1002/cam4.71280

**Published:** 2025-09-29

**Authors:** Jia‐Bei Liu, Qian‐Biao Gu, Jia He, Die‐Juan Liu, Jia‐Lu Long, Hao Li, Peng Liu

**Affiliations:** ^1^ Department of Radiology The First Affiliated Hospital of Hunan Normal University, Hunan Provincial People's Hospital Changsha Hunan Province China; ^2^ Department of Radiology China‐Japan Union Hospital of Jilin University Changchun Jilin Province China

**Keywords:** neutrophil‐to‐lymphocyte ratio, occult liver metastasis, pancreatic ductal adenocarcinoma, predictive model

## Abstract

**Background:**

Pancreatic ductal adenocarcinoma (PDAC) is highly lethal, with liver metastases leading to poorer outcomes. Occult liver metastases (OLM), undetected by initial imaging, complicate treatment and diminish survival rates. We aimed to develop and validate a predictive model for occult liver metastasis in pancreatic cancer, which is crucial for effective preoperative planning.

**Methods:**

A total of 142 patients with PDAC were retrospectively analyzed between January 1, 2020, and December 31, 2023. Malignant cases were confirmed by pathology, and benign cases were confirmed by pathology or follow‐up. Patients were randomly divided into training and validation cohorts at a ratio of 7:3. Factors associated with OLM in PDAC were identified using a stepwise approach, beginning with univariate and followed by multivariate logistic regression analyses. Logistic regression was used to develop clinical, radiological, and combined models, with performance evaluated using the area under the curve (AUC). A nomogram was constructed, and calibration and decision curves were generated. Additionally, machine learning models (RF, SVM, XGBoost) were employed, with AUC and variable importance plots used to evaluate their performance.

**Results:**

Two clinical and four radiological features independently predicted OLM. The combined model achieved an AUC of 0.86 (training) and 0.84 (validation), outperforming clinical (AUC: 0.73, 0.75) and radiological models (AUC: 0.81, 0.75). Machine learning models showed AUCs of 0.787 (RF), 0.850 (SVM), and 0.851 (XGBoost) in the validation cohort. Decision and calibration curves confirmed the combined model's reliability and clinical utility.

**Conclusion:**

The combined model incorporating clinical and radiological features offers a simple, cost‐effective tool to identify PDAC patients at high risk for OLMs, supporting informed surgical decisions and improved outcomes. Integrating clinical and radiological markers enhances early detection and personalized care in PDAC management.

## 1 Introduction

Pancreatic ductal adenocarcinoma (PDAC) is a lethal disease; by 2030, it will be the second‐leading cause of cancer‐related deaths in the United States [1]. In China, the overall 5‐year survival rate for patients is approximately 10% [2]. Although survival rates for many tumors have now improved dramatically, unfortunately, survival rates for PDAC remain stagnant. One of the major reasons is that PDAC is often diagnosed at advanced tumor stages and accompanied by distant metastases. The liver is the most common organ for initial metastatic spread or distant recurrence, which occurs in about 90% of patients with metastatic PDAC [3, 4]. This liver tropism may be explained by the portal venous blood supply and lymphatic drainage, which provide means for hematogenous and lymphatic spread, respectively [5]. Furthermore, patients with PDAC and liver metastases (LMs) have a shorter survival rate than patients with distant metastases at other sites or local recurrence [6]. The 5‐year survival rates for PDAC patients with LMs range from only 2%–5% while those without have a much higher rate [7]. Currently, pathological biopsy remains the gold standard for diagnosis, but it is not feasible for routine screening. Traditional imaging methods have low sensitivity and specificity in detecting small LMs [8, 9]. Preoperative enhanced CT has an accuracy of only 50% for detecting LMs smaller than 1 cm [10, 11, 12], potentially affecting the precision of preoperative staging in PDAC. The detection of LMs remains a significant clinical challenge. Failure to identify LMs can result in suboptimal surgical strategies and adversely impact patient outcomes. Therefore, enhancing the detection of LMs is essential for improving the preoperative management of PDAC.

The liver is the most common site for distant metastasis in PDAC and represents a significant risk factor for poor prognosis. Despite the growing use of MRI and PET/CT, CT remains the most widely employed tool for detection and evaluation. However, the limited sensitivity of CT can result in missed small metastases, with approximately 10% of resectable or borderline‐resectable PDAC patients showing suspicious liver or peritoneal metastases during surgical exploration [13, 14, 15]. Occult metastasis (OM) refers to metastatic lesions undetectable on preoperative imaging but discovered intraoperatively. Patients with metastasis are ineligible for curative surgery and must receive systemic therapy instead, such as chemotherapy [16, 17]. Thus, early identification of LMs is critical for selecting optimal treatment strategies and improving patient outcomes.

Although radiomics has shown potential in predicting LMs in pancreatic cancer in recent years [18, 19], its widespread clinical application is limited by its high cost and reliance on advanced image analysis techniques. Moreover, the complexity of radiomics models hinders their interpretability and ease of use in clinical decision‐making. In this context, our study aims to develop a predictive model based on preoperative clinical and subjective imaging data to improve the accuracy of predicting occult LMs in pancreatic cancer. Compared to radiomics‐based approaches, the advantages of our method lie in its simplicity, low cost, and broad applicability. Our approach does not require complex image processing software, making it easy to implement in various healthcare settings while providing clinicians with intuitive and easily interpretable predictive results. Additionally, by integrating clinical and imaging data, our model offers more comprehensive predictive information, aiding physicians in making more accurate preoperative treatment decisions.

While previous studies have revealed that the circulating tumor cell (CTC) count is a significant predictor of LMs within 6 months postoperatively [20], intraoperatively collected portal vein CTC counts cannot prevent unnecessary surgeries and serve only as a guide for adjuvant therapy. These findings are often limited to laboratory settings and are challenging to translate into clinical practice. In contrast, our model is based on real‐world clinical data, making it more clinically applicable and valuable for prediction. Similarly, although contrast‐enhanced ultrasound (CEUS) is a non‐invasive, radiation‐free imaging technique with potential for predicting occult LMs in pancreatic cancer [21], its clinical use has limitations. These include reduced sensitivity for detecting small metastases (< 10 mm) and poor efficacy in identifying peritoneal micro‐metastases [22]. CEUS accuracy is also operator‐dependent, requiring specialized knowledge and experience, posing challenges for less experienced practitioners [23]. Furthermore, factors such as lesion location, patient body habitus, and gastrointestinal gas may affect image clarity and diagnostic accuracy [24].

In summary, this study aims to develop a simple, cost‐effective, easy‐to‐implement predictive model for occult LMs in PDAC by combining preoperative clinical and subjective imaging data. Our research not only addresses the limitations of existing studies but also provides new perspectives and tools for the clinical management and treatment of pancreatic cancer.

## 2 Materials and Methods

### 2.1 Study Population

**FIGURE 1:**
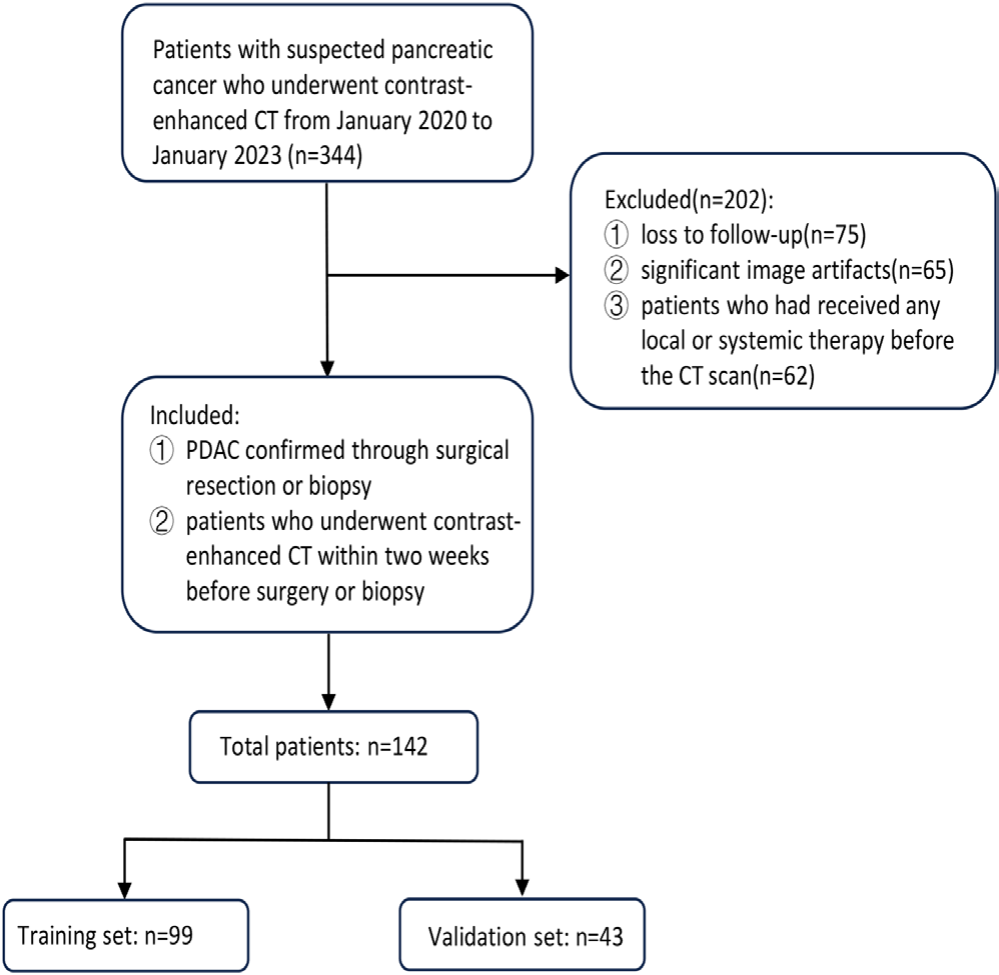
Flowchart of the patient selection process.

This study was approved by the institutional ethics review board, and the requirement for informed consent was waived due to its retrospective design. A total of 142 patients with pathologically confirmed PDAC were included from Hunan Normal University First Affiliated Hospital and Hunan Provincial People's Hospital between January 1, 2020, and December 31, 2023 (Figure 1). The inclusion criteria were: (1) patients with PDAC confirmed through surgical resection or biopsy; (2) patients who underwent contrast‐enhanced CT within 2 weeks before surgery or biopsy; and (3) patients who had not received any local or systemic therapy before the CT scan. The exclusion criteria were: (1) loss to follow‐up, defined as the absence of clinical or imaging data after the initial diagnosis; and (2) significant image artifacts (e.g., motion artifacts or poor contrast quality). Patients were randomly assigned to the training and validation cohorts in a 7:3 ratio using a random number generator. Occult LMs were defined as liver metastases that were either undetectable on preoperative CT imaging, had a diameter of less than 1 cm on CT, or were identified intraoperatively or within 3 months postoperatively through follow‐up imaging or clinical evaluation.

### 2.2 Clinical Variables Collection

Comprehensive demographic and clinical data were collected within a 2‐week period prior to surgery. This information included basic patient characteristics such as age and sex, along with tumor‐related biomarkers: carbohydrate antigen 199 (CA199), carbohydrate antigen 125 (CA125), and carbohydrate antigen 724 (CA724). Additionally, liver function indicators were assessed, comprising alkaline phosphatase (ALP), alanine transaminase (ALT), aspartate transaminase (AST), and total bilirubin level (TBIL). Nutritional and hematological parameters included albumin, absolute neutrophil count, absolute lymphocyte count, and platelet count. These data were extracted from the electronic medical records, ensuring accuracy and consistency.

Inflammatory indices, which play a significant role in cancer prognosis, were calculated as follows: the Neutrophil‐to‐Lymphocyte Ratio (NLR) as the absolute neutrophil count divided by the absolute lymphocyte count, the Platelet‐to‐Lymphocyte Ratio (PLR) as the absolute platelet count divided by the absolute lymphocyte count, and the Systemic Immuno‐Inflammation Index (SII) as the product of the platelet count and NLR. The optimal cutoff values for SII, NLR, and PLR were derived from the receiver operating characteristic (ROC) curve analysis in the training cohort and were subsequently validated in the validation cohort. Patients were subsequently divided into the following groups: low SII (≤ 694) and high SII (> 694); low PLR (≤ 125) and high PLR (> 125); low NLR (≤ 4) and high NLR (> 4).

### 2.3 CT Examination and Variables

All patients were instructed to fast from solid food for approximately 4–6 h prior to the examinations. Given the extended duration of data collection, multiple CT scanners were employed, including the Siemens Force, UCT960, and Philips 256‐slice Brilliance iCT scanner. Enhanced CT imaging was performed during the plain, arterial, and portal venous phases. The CT images were acquired using a protocol set at 120 kVp with a range of 150–350 mAs, a slice thickness between 0.5 and 1 mm, and a field of view ranging from 320 to 380 mm. A nonionic contrast medium (Ultravist/iopromide 370; Omnipaque 300 g/L; 80–100 mL) was administered intravenously at a rate of 3–5 mL/s for the enhanced CT images. The arterial phase and portal venous phase images were captured at 28–30 and 45–60 s, respectively.

CT variables were selected based on the parameters outlined in the PDAC radiology reporting template proposed by the Society of Abdominal Radiology and the American Pancreatic Association [25]. These variables included dominant tumor location, size, and tumor density compared with surrounding parenchyma (i.e., hypo‐, iso‐, or hyperdense) during both the arterial and portal venous phases. Other important variables included tumor contact with the portal vein or superior mesenteric vein, suspicious metastatic lymph nodes, and evidence of adjacent organ invasion. All variables were evaluated according to the definitions in the reporting template.

Additional CT variables assessed were vein thrombus, pancreatic duct dilatation, biliary dilatation, splenic vein involvement, collateral circulation, tumor conspicuity (defined as well [clearly delineated tumor margin], moderate [visible tumor but indistinct margin], or poor [indistinct tumor]), and tumor necrosis (characterized by non‐enhancement in the arterial phase and portal venous phase) [26]. The Arterial Enhancement Fraction (AEF), which assesses lesion blood supply, was calculated as the difference in CT density between the arterial and non‐contrast phases divided by the difference between the portal venous and non‐contrast phase values, expressed as a percentage. Based on the cutoff value determined from the training set, AEF was classified into two groups: ≥ 0.57 and < 0.57.

All these variables were evaluated by consensus between two radiologists (LJB and GQB), each with 1 year of experience in pancreatic imaging. Both radiologists were blinded to the postsurgical outcome data. Intraclass correlation coefficients (ICCs) of the assessed features were calculated, with values exceeding 0.75 indicating good reliability.

### 2.4 Statistical Analysis

Data were analyzed using R software (version 4.4.0). Quantitative variables were summarized by median values and ranges, while categorical variables were reported as frequencies and percentages. Training and validation cohorts were randomly assigned using R's sample() function with a predefined ratio to ensure an unbiased distribution. Univariate and multivariate logistic regression analyses were then conducted to examine the impact of selected indicators on patient prognosis, with Odds Ratios (OR) and 95% confidence intervals (CI) calculated for precision. Three predictive models were constructed using logistic regression: clinical, radiological, and combined models. Additionally, the combined model was built with three classifiers, Random Forest (RF), Support Vector Machine (SVM), and Gradient Boosting (XGBoost). RF employs an ensemble learning approach, which integrates predictions from multiple decision trees during training and ultimately classifies samples by majority voting. SVM operates as a supervised learning algorithm that identifies the optimal hyperplane in a high‐dimensional space to maximize the separation between different classes. XGBoost, short for Extreme Gradient Boosting, is an efficient and scalable implementation of XGBoost machines that enhances predictive performance and computational efficiency through advanced regularization and parallel processing. Hyperparameter optimization was carried out using 10‐fold cross‐validation to ensure reproducibility. ROC curves were constructed to determine the optimal cutoff values for SII, PLR, and NLR, maximizing the Youden index. The area under the curve (AUC) of the ROC was used to evaluate the model's predictive performance. Decision curve analysis (DCA) was employed to assess the clinical utility of the nomogram, providing insight into its decision‐making implications. Statistical significance was set at *p* < 0.05, and adjustments for multiple comparisons were considered as necessary. Furthermore, we used variable importance plots to interpret the predictors in the three machine learning models, providing insight into feature importance and model interpretation.

## 3 Results

### 3.1 Characteristics of Study Population

The baseline clinical and radiological characteristics of the training and validation cohorts are shown in Tables 1 and 2. We included 142 patients [mean age 60.8 ± 9 years, 89 (62.68%) male], among whom 65 patients had occult LMs (45.77%), and 77 patients without occult LMs (54.23%). There were no significant differences observed in clinical features between the training cohort and validation cohort.

**TABLE 1:** Clinical characteristics of patients.

Characteristic	ALL *N* = 142	Test *N* = 43	Train *N* = 99	*p*
Gender				
Female	53 (37.32%)	15 (34.88%)	38 (38.38%)	0.836
Male	89 (62.68%)	28 (65.12%)	61 (61.62%)	
Age (years)	60.80 (±9.00)	61.16 (±10.12)	60.64 (±8.52)	0.766
CA199 (U/mL)				
≤ 37	26 (18.31%)	11 (25.58%)	15 (15.15%)	0.215
> 37	116 (81.69%)	32 (74.42%)	84 (84.85%)	
CA125 (U/mL)				
≤ 35	85 (59.86%)	23 (53.49%)	62 (62.63%)	0.404
> 35	57 (40.14%)	20 (46.51%)	37 (37.37%)	
CA724 (U/mL)				
≤ 6	92 (64.79%)	29 (67.44%)	63 (63.64%)	0.806
> 6	50 (35.21%)	14 (32.56%)	36 (36.36%)	
Total bilirubin (μmol/L)				
≤ 17	64 (45.07%)	23 (53.49%)	41 (41.41%)	0.252
> 17	78 (54.93%)	20 (46.51%)	58 (58.59%)	
Albumin (g/L)				
≤ 35	16 (11.27%)	2 (4.65%)	14 (14.14%)	0.148
> 35	126 (88.73%)	41 (95.35%)	85 (85.86%)	
ALT (U/L)				
≤ 50	73 (51.41%)	26 (60.47%)	47 (47.47%)	0.215
> 50	69 (48.59%)	17 (39.53%)	52 (52.53%)	
AST (U/L)				
≤ 40	73 (51.41%)	26 (60.47%)	47 (47.47%)	0.215
> 40	69 (48.59%)	17 (39.53%)	52 (52.53%)	
ALP (U/L)				
≤ 125	76 (53.52%)	27 (62.79%)	49 (49.49%)	0.202
> 125	66 (46.48%)	16 (37.21%)	50 (50.51%)	
Neutrophils (×10^9^)	3.84 [2.99; 5.06]	3.84 [2.86; 5.32]	3.84 [3.13; 4.90]	0.905
Lymphocytes (×10^9^)	1.13 [0.97; 1.47]	1.16 [1.02; 1.47]	1.13 [0.96; 1.44]	0.537
Platelets (×10^9^)	219.00 [160.25; 269.75]	206.00 [153.00; 243.50]	221.00 [168.00; 274.50]	0.268
NLR				
≤ 4	78 (54.93%)	23 (53.49%)	55 (55.56%)	0.965
> 4	64 (45.07%)	20 (46.51%)	44 (44.44%)	
PLR				
≤ 125	34 (23.94%)	11 (25.58%)	23 (23.23%)	0.930
> 125	108 (76.06%)	32 (74.42%)	76 (76.77%)	
SII				
≤ 694	67 (47.18%)	24 (55.81%)	43 (43.43%)	0.240
> 694	75 (52.82%)	19 (44.19%)	56 (56.57%)	
Occult liver metastases	65 (45.77%)	22 (51.16%)	43 (43.43%)	0.403

*Note:* Data in square brackets (方括号) are 95% confidence intervals.

Abbreviations: ALP, alkaline phosphatase; ALT, alanine transaminase; AST, aspartate transaminase; NLR, neutrophil‐to‐lymphocyte ratio; PLR, platelet‐to‐lymphocyte ratio; SII, systemic immune inflammation index.

**TABLE 2:** Radiological characteristics of patients.

Characteristic	ALL *N* = 142	Test *N* = 43	Train *N* = 99	*p*
Tumor location				
Body and tail	44 (30.99%)	14 (32.56%)	30 (30.30%)	0.945
Head and neck	98 (69.01%)	29 (67.44%)	69 (69.70%)	
Tumor size (cm)				
≤ 4	120 (84.51%)	37 (86.05%)	83 (83.84%)	0.935
> 4	22 (15.49%)	6 (13.95%)	16 (16.16%)	
Tumor density in AP				
Isodense or hyperdense	126 (88.73%)	37 (86.05%)	89 (89.90%)	0.567
Hypodense	16 (11.27%)	6 (13.95%)	10 (10.10%)	
Tumor density in PVP				
Isodense or hyperdense	128 (90.14%)	39 (90.70%)	89 (89.90%)	1.000
Hypodense	14 (9.86%)	4 (9.30%)	10 (10.10%)	
Tumor conspicuity in AP				
Poor	34 (23.94%)	12 (27.91%)	22 (22.22%)	0.629
Moderate	61 (42.96%)	19 (44.19%)	42 (42.42%)	
Well	47 (33.10%)	12 (27.91%)	35 (35.35%)	
Tumor conspicuity in PVP				
Poor	24 (16.90%)	9 (20.93%)	15 (15.15%)	0.347
Moderate	77 (54.23%)	25 (58.14%)	52 (52.53%)	
Well	41 (28.87%)	9 (20.93%)	32 (32.32%)	
Contact to SMV or PV				
No	88 (61.97%)	24 (55.81%)	64 (64.65%)	0.419
Yes	54 (38.03%)	19 (44.19%)	35 (35.35%)	
Tumor necrosis				
No	78 (54.93%)	21 (48.84%)	57 (57.58%)	0.437
Yes	64 (45.07%)	22 (51.16%)	42 (42.42%)	
Suspicious metastatic lymph nodes				
No	103 (72.54%)	31 (72.09%)	72 (72.73%)	1.000
Yes	39 (27.46%)	12 (27.91%)	27 (27.27%)	
Adjacent organ invasion				
No	102 (71.83%)	27 (62.79%)	75 (75.76%)	0.169
Yes	40 (28.17%)	16 (37.21%)	24 (24.24%)	
Venous thrombus				
No	138 (97.18%)	42 (97.67%)	96 (96.97%)	1.000
Yes	4 (2.82%)	1 (2.33%)	3 (3.03%)	
Pancreatic duct dilatation				
No	49 (34.51%)	13 (30.23%)	36 (36.36%)	0.607
Yes	93 (65.49%)	30 (69.77%)	63 (63.64%)	
Biliary dilatation				
No	74 (52.11%)	20 (46.51%)	54 (54.55%)	0.485
Yes	68 (47.89%)	23 (53.49%)	45 (45.45%)	
Spleen involvement				
No	111 (78.17%)	32 (74.42%)	79 (79.80%)	0.623
Yes	31 (21.83%)	11 (25.58%)	20 (20.20%)	
Collateral circulation				
No	131 (92.25%)	40 (93.02%)	91 (91.92%)	1.000
Yes	11 (7.75%)	3 (6.98%)	8 (8.08%)	
AEF				
< 0.57	76 (53.52%)	21 (48.84%)	55 (55.56%)	0.579
≥ 0.57	66 (46.48%)	22 (51.16%)	44 (44.44%)	

Abbreviations: AEF, Arterial Enhancement Fraction; AP, arterial phase; PV, portal vein; PVP, portal venous phase; SMV, superior mesenteric vein.

### 3.2 Clinical Factors Associated With Occult LMs

Analysis of the clinical features in the training group demonstrated that male gender, CA125 > 35 U/mL, and a higher NLR were risk factors for occult liver metastases (OLM). Table 3 multivariate analysis showed that CA125 > 35 U/mL (OR: 2.66; 95% CI, 1.09–6.69) and higher NLR (OR: 4.45; 95% CI, 1.80–11.64) were independent risk factors for occult LMs.

**TABLE 3:** Univariate and multivariate logistic regression analyses of predictors for occult liver metastasis of PDAC (clinical).

Characteristics	Univariate analysis		Multivariate analysis	
	OR (95% CI)	*p*	OR (95% CI)	*p*
Gender	2.708 (1.165–6.607)	0.024*	2.550 (0.982–6.990)	0.059
Age	1.012 (0.965–1.062)	0.622		
CA199 (U/mL)	0.857 (0.282–2.652)	0.784		
CA125 (U/mL)	4.178 (1.791–10.17)	0.001*	4.444 (1.802–11.64)	0.002*
CA724 (U/mL)	1.52 (0.666–3.494)	0.32		
Total bilirubin (μmol/L)	0.582 (0.257–1.305)	0.19		
Albumin (g/L)	1.455 (0.462–5.073)	0.531		
ALT (U/L)	0.552 (0.244–1.226)	0.147		
AST (U/L)	0.652 (0.291–1.447)	0.295		
ALP (U/L)	0.638 (0.284–1.416)	0.272		
Neutrophils (×10^9^)	0.965 (0.791–1.158)	0.708		
Lymphocytes (×10^9^)	0.66 (0.269–1.534)	0.344		
Platelets (×10^9^)	0.999 (0.995–1.004)	0.827		
NLR	3.229 (1.426–7.542)	0.006*	2.660 (1.088–6.686)	0.034*
PLR	2.057 (0.783–5.877)	0.156		
SII	1.571 (0.702–3.577)	0.275		

* *p* < 0.05 was considered statistically significant.

### 3.3 Radiological Factors Associated With LMs

Univariate analysis revealed that tumor location, tumor size, necrosis, suspicious metastatic lymph nodes, adjacent organ invasion, and splenic vein involvement were risk factors for occult LMs. Table 4 multivariate analysis demonstrated that splenic vein involvement (OR: 3.75; 95% CI, 1.06–14.45), necrosis (OR: 4.68; 95% CI, 1.75–13.74), suspicious metastatic lymph nodes (OR: 6.72; 95% CI, 2.17–23.65), and adjacent organ invasion (OR: 4.30; 95% CI, 1.30–15.45) were independent risk factors for LMs.

**TABLE 4:** Univariate and multivariate logistic regression analyses of radiological predictors for occult liver metastasis of PDAC (radiological).

Characteristics	Univariate analysis		Multivariate analysis	
	OR (95% CI)	*p*	OR (95% CI)	*p*
Tumor location	0.25 (0.097–0.608)	0.3		
Tumor size	5.032 (1.598–19.26)	0.009*	2.947 (0.674–14.54)	0.16
Tumor density in AP	0.525 (0.108–2.022)	0.372		
Tumor density in PVP	0.293 (0.043–1.248)	0.133		
Tumor conspicuity in AP	0.886 (0.518–1.51)	0.655		
Tumor conspicuity in PVP	1.402 (0.77–2.609)	0.274		
Tumor necrosis	3.824 (1.671–9.073)	0.002*	4.680 (1.748–13.74)	0.003*
Contact to SMV or PV	1.979 (0.862–4.618)	0.109		
Suspicious metastatic lymph nodes	4.75 (1.873–13.01)	0.001*	6.723 (2.171–23.65)	0.002*
Adjacent organ invasion	3.556 (1.38–9.803)	0.01*	4.298 (1.308–15.45)	0.019*
Venous thrombus	2.48 (1.00–6.19)	0.51		
Pancreatic duct dilatation	0.551 (0.238–1.258)	0.158		
Biliary dilatation	0.654 (0.289–1.456)	0.301		
Spleen involvement	4.023 (1.447–12.43)	0.01*	3.752 (1.064–14.45)	0.044*
Collateral circulation	2.325 (0.538–11.89)	0.267		
AEF	1.37 0.615–3.068)	0.441		

* *p* < 0.05.

### 3.4 Construction and Performance Verification of the Prediction Model

**FIGURE 2:**
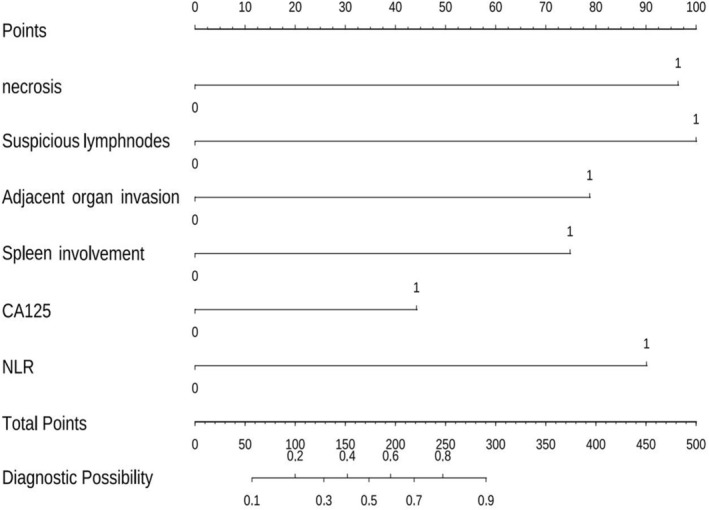
Nomogram model based on CA125, NLR, tumor necrosis, suspicious metastatic lymph nodes, splenic vein involvement, and adjacent organ invasion.

**FIGURE 3:**
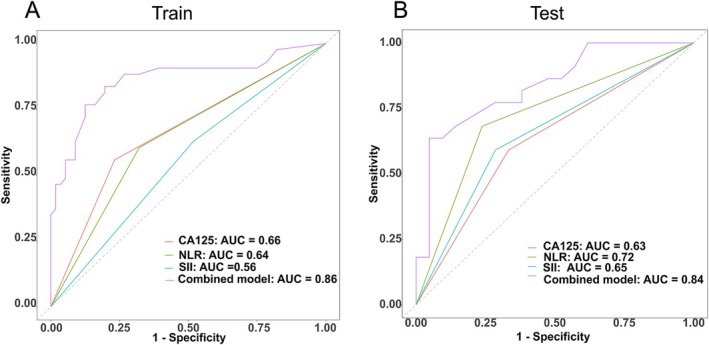
The AUC values for CA125, NLR, SII, and the combined model. (A) AUC for the training cohort. (B) AUC for the validation cohort. Notably, the combined model demonstrated a significantly higher AUC compared to each individual marker, indicating its superior predictive performance.

**FIGURE 4:**
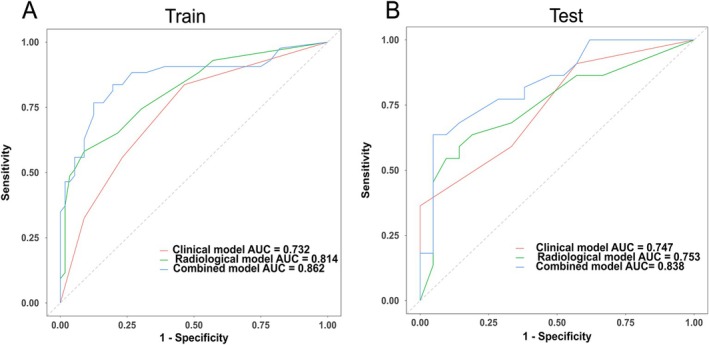
AUC of the clinical model, radiological model, and combined model. (A) AUC for the training cohort. (B) AUC for the validation cohort.

**FIGURE 5:**
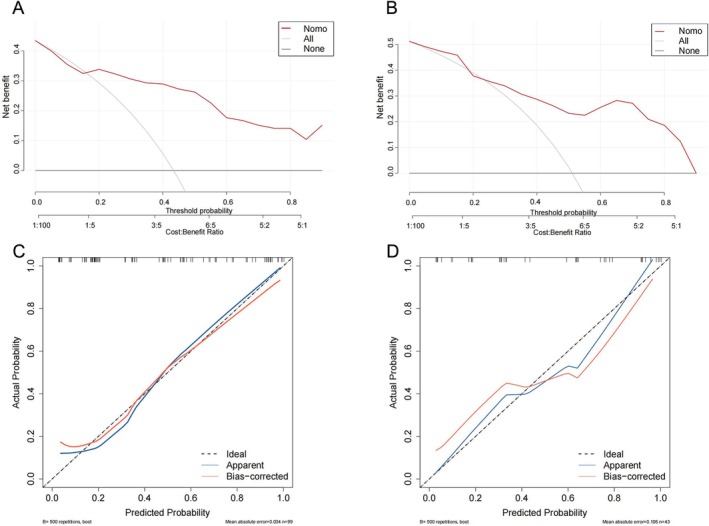
Comparison of the decision and calibration curves. (A) Decision curve for the training cohort; (B) decision curve for the validation cohort; (C) calibration curve for the training cohort; (D) calibration curve for the validation cohort.

**FIGURE 6:**
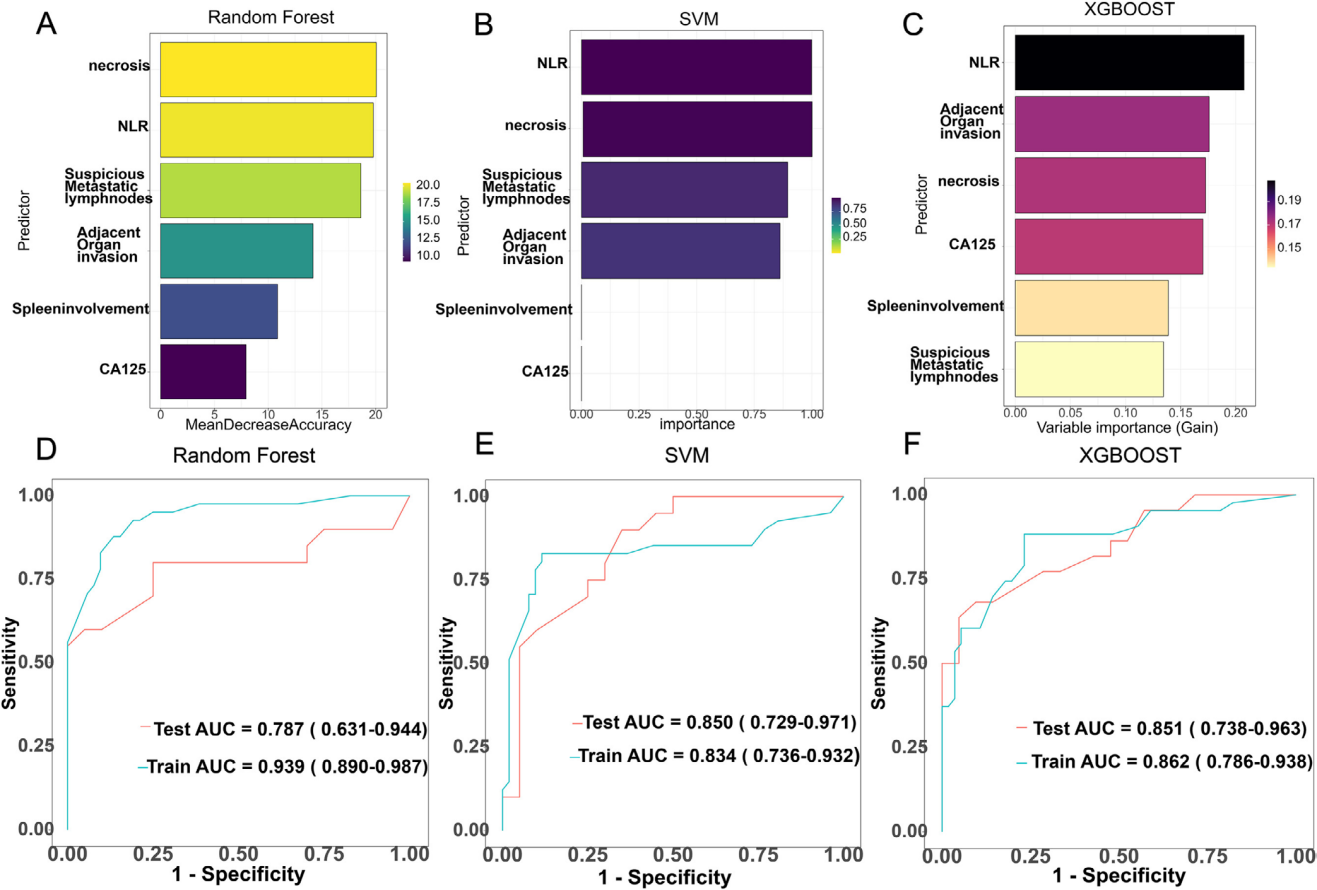
Variable importance and AUC of three machine learning models. (A) Random Forest (RF) variable importance measured by mean decrease in accuracy. (B) Support Vector Machine (SVM) variable importance based on the absolute coefficients of the linear kernel. (C) XGBoost variable importance measured by gain. (D–F) AUC of the RF, SVM, and XGBoost models in the training and validation sets.

We incorporated the identified independent predictive factors into a model and constructed a visual nomogram to facilitate risk assessment, as shown in Figure 2. The combined model achieved an AUC of 0.84, outperforming CA125 (AUC = 0.63), NLR (AUC = 0.72), and SII (AUC = 0.65). These results highlight the advantages of integrating clinical and radiological features in a predictive model (Figure 3). The ROC curves for the training and validation groups of logistic regression are presented in Figure 4. In the training group, the combined model demonstrated the highest AUC of 0.86 (95% CI: 0.78–0.94). The AUCs for the clinical model and the radiological model were 0.73 (95% CI: 0.64–0.83) and 0.81 (95% CI: 0.73–0.90), respectively. Similar predictive accuracy was observed in the validation cohort, with AUC values of 0.84 (95% CI: 0.72–0.96) for the combined model, and 0.75 (95% CI: 0.61–0.89) and 0.75 (95% CI: 0.60–0.90) for the clinical and radiological models, respectively. Furthermore, DCA revealed that the nomogram provided clinical benefits for both groups, as demonstrated in Figure 5A,B. Robust correlations were observed between the predicted and actual outcomes in the calibration curves for both the training and validation groups, as shown in Figure 5C,D. Additionally, Figure 6 highlights the AUCs of the RF, SVM, and XGBoost, and the variable importance plots, respectively. The DeLong test indicated no statistically significant differences (all *p* > 0.05).

## 4 Discussion

Our study developed and validated a predictive model for OLM in PDAC patients by integrating key clinical and CT imaging features. The most significant predictors identified were elevated CA125, a high NLR, tumor necrosis, suspicious metastatic lymph nodes, splenic vein involvement, and adjacent organ invasion.

The combined model demonstrated superior predictive performance compared to models using clinical or radiological features alone, achieving an AUC of 0.85. This outperformed established single markers such as CA125 (AUC = 0.73; Liu et al., 2016) and SII (AUC = 0.70; Han et al., 2022) [27, 28], as summarized in Table 5. The integration of radiological characteristics—which provide direct visual evidence of tumor morphology and local invasion—with systemic inflammatory and tumor markers offers a more comprehensive assessment of metastatic risk. This aligns with emerging radiomics‐based approaches [18], but our reliance on routinely available subjective imaging assessments and clinical biomarkers enhances its practicality for real‐world clinical settings.

**TABLE 5:** Comparison of predictive performance (AUC) between the current combined model and previously reported biomarkers for outcomes in pancreatic ductal adenocarcinoma.

Study	Population/outcome	Marker	AUC
Liu et al. (2016)	Prediction of metastasis	CA125	0.73
Han et al. (2022)	Prognosis in metastatic PDAC	SII	0.70
Current Study	Prediction of occult liver metastasis	Combined model	0.85

To ensure analytical robustness, we employed multiple machine learning algorithms, including RF, SVM, XGBoost, and logistic regression. While ensemble methods such as XGBoost and SVM also showed high predictive accuracy (AUCs: 0.787–0.851), the logistic regression model was selected for final implementation due to its favorable balance of performance and interpretability—a key consideration for clinical adoption.

A previous study has suggested that preoperative tumor marker CA125 ≥ 30 IU/mL was an independent risk factor for early LMs [29], which was similar to our study. In our study, significant differences were observed in preoperative CA125 levels, which were identified as an independent predictor of occult LMs in PDAC patients. Elevated preoperative CA125 levels indicate aggressive tumor characteristics and the possibility of micro‐metastasis [30]. Previous studies have established CA125 as a definitive biomarker for ovarian cancer, where it is associated with peritoneal metastasis through mediating cell adhesion [31]. However, other research has found that elevated serum CA125 levels (> 35 U/mL) in patients with pancreatic ductal adenocarcinoma (PDAC) are associated with a higher recurrence rate and increased distant organ metastasis [32, 33].

Increasing evidence suggests that immune status and inflammatory and nutritional status affect the prognosis of malignant tumors. In addition, these immunoinflammatory cells also contribute to tumor cell extravasation, survival in peripheral blood, and subsequent distant metastasis of tumor cells [34].

As expected, our data show that NLR plays a key role in the prediction of OLM in PDAC. NLR is a systemic inflammatory response marker associated with the prognosis of various malignant tumors. A high NLR primarily reflects an increase in neutrophils and a decrease in lymphocytes, indicating a weakened immune response and heightened inflammatory activity in PDAC patients [35, 36, 37]. This imbalance is associated with poor prognosis and may be linked to the occurrence of occult LMs. In Peng's study [34], they found NLR was an independent prognostic factor of OS in patients with pancreatic cancer liver metastasis (PCLM); then they developed a nomogram, which, based on immune, inflammation, nutritional status, and other clinical factors, can accurately predict OS of PCLM patients. This finding aligns with our research outcomes, suggesting a consensus within the field regarding the significance of NLR in PCLM patients.

In contrast to Aziz's findings [38], however, their research demonstrated that SIII is an independent predictor of cancer‐specific survival and recurrence in patients with resectable PDAC. Our study, on the other hand, focuses on the role of NLR in occult LMs of pancreatic cancer, suggesting that different biomarkers may play distinct roles at various stages and in different subtypes of the disease. A possible explanation for this might be that higher NLR values were associated with worse survival outcomes, suggesting that elevated systemic inflammation and impaired immune surveillance contribute to the progression of LMs and poorer prognosis in these patients. This finding highlights the potential clinical value of NLR as a biomarker for predicting occult LMs in PDAC. The presence of higher NLR was observed in over half (63.1%) of occult LMs of PDAC in our research, suggesting that higher NLR could serve as a robust predictor in clinical practice.

In this study, we employed subjective imaging features, which not only enhance clinical feasibility through seamless integration into routine practice but also provide superior visualization compared to quantitative metrics or radiomics features commonly used in other studies. Our interesting finding is that tumor necrosis, suspicious metastatic lymph nodes, splenic vein involvement, and adjacent organ invasion are independent predictors of occult LMs in PDAC. The identification of these imaging features is significant for preoperative assessment and the formulation of treatment strategies.

Based on prior studies, tumor necrosis may be associated with tumor aggressiveness and the inflammatory state of the microenvironment [39, 40, 41]. In PDAC, tumor necrosis may promote the shedding of tumor cells and distant metastasis. Necrotic areas of the tumor may release factors that facilitate tumor growth and metastasis, such as vascular endothelial growth factor (VEGF) and tumor necrosis factor‐alpha (TNF‐α) [42]. The presence of suspicious metastatic lymph nodes may indicate that the tumor has already spread via the lymphatic pathway. Lymph node metastasis is a critical route for tumor dissemination, and the metastatic status of lymph nodes can serve as an important indicator of tumor aggressiveness and potential risk of distant metastasis.

Tumor necrosis and suspicious metastatic lymph nodes, as demonstrated in Kim's study, exhibited notable predictive performance for recurrence or death after surgery in PDAC patients [43]. A significant disparity in tumor necrosis and suspicious metastatic lymph nodes was also observed between the occult LMs and non‐occult LMs groups in our study. Additionally, we found that splenic vein involvement and adjacent organ invasion are independent predictors of occult LMs in pancreatic cancer. These findings reflect the extent of the tumor and suggest an increased likelihood of tumor cells entering the circulatory system, thereby elevating the risk of distant metastasis.

While our study offers valuable insights, it has several limitations. First, the relatively small sample size is a limitation of this study and may affect the generalizability of the findings. Despite this, the consistent model performance across the training and validation datasets supports the reliability of the observed results. Second, being a single‐center study, there is potential for selection bias in the patient cohort, which could affect the external validity of the results. Additionally, the retrospective nature of data collection may introduce information bias, particularly when subjective imaging assessments are involved. Future research should employ a prospective, multicenter design with a larger sample size to improve the robustness and generalizability of the findings. Furthermore, the potential confounding effect of preoperative biliary stenting was not accounted for in our analysis, as our dataset comprised solely of pre‐interventional data. Moreover, further studies should evaluate the applicability of these predictive factors in diverse populations and across various stages of pancreatic cancer, as well as explore how these factors can be integrated with emerging biomarkers and radiomics technologies to enhance predictive accuracy. Finally, these limitations highlight the need for future prospective, multicenter studies to validate our model's robustness and clinical applicability in larger, more diverse cohorts.

## 5 Conclusion

Integrating radiological and clinical features significantly predicts OLM in PDAC patients. Key predictors—CA125, NLR, tumor necrosis, metastatic lymph nodes, splenic vein involvement, and organ invasion—enhance our understanding of PDAC metastasis and inform preoperative assessments, guiding personalized treatment strategies. Incorporating these factors into clinical practice could optimize patient stratification for occult LMs risk.

## Author Contributions

**Jia‐Bei Liu**, and **Qian‐Biao Gu:** contributed to manuscript writing and editing. **Peng Liu:** contributed to conceptualization and supervision. All authors have read and approved the final manuscript.

## Ethics Statement

The retrospective study, approved by the ethics committee of The First Affiliated Hospital of Hunan Normal University (Hunan Provincial People's Hospital) and fully adhered to the Declaration of Helsinki, waived the requirement of written informed consent.

## Conflicts of Interest

The authors declare no conflicts of interest.

## Data Availability

The data that support the findings of this study are available from the corresponding author upon reasonable request.
